# WHO/ISUP grading of clear cell renal cell carcinoma and papillary renal cell carcinoma; validation of grading on the digital pathology platform and perspectives on reproducibility of grade

**DOI:** 10.1186/s13000-021-01130-2

**Published:** 2021-08-21

**Authors:** Lisa Browning, Richard Colling, Clare Verrill

**Affiliations:** 1 Department of Cellular Pathology, Oxford University Hospitals NHS Trust, John Radcliffe Hospital, Headley Way, OX3 9DU Oxford, UK; 2 NIHR Oxford Biomedical Research Centre, Oxford University Hospitals NHS Foundation Trust, Oxford, Oxfordshire UK; 3 Nuffield Department of Surgical Sciences, University of Oxford, John Radcliffe Hospital, OX3 9DU Oxford, UK

**Keywords:** Validation, Renal carcinoma, ISUP, Grading, Digital pathology, Reproducibility

## Abstract

**Background:**

There are recognised potential pitfalls in digital diagnosis in urological pathology, including the grading of dysplasia. The World Health Organisation/International Society of Urological Pathology (WHO/ISUP) grading system for renal cell carcinoma (RCC) is prognostically important in clear cell RCC (CCRCC) and papillary RCC (PRCC), and is included in risk stratification scores for CCRCC, thus impacting on patient management. To date there are no systematic studies examining the concordance of WHO/ISUP grading between digital pathology (DP) and glass slide (GS) images. We present a validation study examining intraobserver agreement in WHO/ISUP grade of CCRCC and PRCC.

**Methods:**

Fifty CCRCCs and 10 PRCCs were graded (WHO/ISUP system) by three specialist uropathologists on three separate occasions (DP once then two GS assessments; GS1 and GS2) separated by wash-out periods of at least two-weeks. The grade was recorded for each assessment, and compared using Cohen’s and Fleiss’s kappa.

**Results:**

There was 65 to 78% concordance of WHO/ISUP grading on DP and GS1. Furthermore, for the individual pathologists, the comparative kappa scores for DP versus GS1, and GS1 versus GS2, were 0.70 and 0.70, 0.57 and 0.73, and 0.71 and 0.74, and with no apparent tendency to upgrade or downgrade on DP versus GS. The interobserver kappa agreement was less, at 0.58 on DP and 0.45 on GS.

**Conclusion:**

Our results demonstrate that the assessment of WHO/ISUP grade on DP is noninferior to that on GS. There is an apparent slight improvement in agreement between pathologists on RCC grade when assessed on DP, which may warrant further study.

## Introduction

Adoption of digital pathology (DP) into clinical diagnostic practice is still in its early stages. In our department we now scan 100 % of the surgical histology slides, and the digital imaging of urological pathology cases has been routine since January 2019. The urological pathologists have undergone a validation process to facilitate safe digital reporting in the specialty, in accordance with guidance from the Royal College of Pathologists [1]. Whilst there are multiple large validation studies which provide evidence that DP is non-inferior to light microscopy diagnosis [2–10], including specifically for remote DP reporting [11–13] there remain recognised ‘pitfalls’ of digital diagnosis that need to be acknowledged, one of which is grading of dysplasia [1, 14]. In urological pathology specifically, the World Health Organisation/International Society of Urological Pathology (WHO/ISUP) grading of renal cell carcinoma (RCC) digitally has been identified by one group as a feature of potential challenge [14].

The WHO/ISUP grading system is validated for use in clear cell renal cell carcinoma (CCRCC) and papillary renal cell carcinoma (PRCC), but not chromophobe renal cell carcinoma, or other renal tumour types. Grading is based upon nucleolar prominence (grades 1–3) and with grade 4 tumours showing nuclear anaplasia +/- giant cells, rhabdoid and/or sarcomatoid features. This system is based upon evidence that has shown that patient outcome in CCRCC and PRCC is correlated with the prominence of tumour cell nucleoli [15, 16]. Importantly WHO/ISUP grade is of prognostic significance and is incorporated into patient risk stratification systems such as the Leibovich score [17] which can be used to predict likely progression to metastatic disease in patients with localised CCRCC, thereby influencing patient follow-up, including potential clinical trial entry.

Published primary studies specifically investigating concordance in WHO/ISUP grading of RCC between DP and glass slides (GS) are however lacking. We have therefore undertaken a study to determine the concordance of WHO/ISUP grade assigned to CCRCC and PRCC on DP and GS, which includes the baseline assessment of the intraobserver concordance of WHO/ISUP grading on GS. This validation study design is in accordance with guidance from the College of American Pathology and Laboratory Quality Center [18], and in line with other published validation studies [2]. Whilst not specifically a requirement for a DP validation study, we also determined the interobserver agreement on WHO/ISUP grade for these cases.

## Methods

The histopathology database in our department was searched for CCRCC and PRCC cases (nephrectomy, partial nephrectomy, biopsy) reported during 2019. These cases had been scanned at x40 magnification on the Philips IntelliSite® Ultrafast Scanner as part of the routine workflow in the department for uropathology since January 2019. Consecutive cases of CCRCC and of PRCC were assessed to ensure that the digital images were of appropriate quality for diagnostic assessment; out of focus cases and those with other artefacts were excluded. From the search, 50 CCRCCs and 10 PRCCs were identified, and two H&E sections of tumour from each resection case were selected, with one H&E section available for the biopsy cases. The study was registered within our centre as a clinical audit and did not require specific ethics approval.

Three specialist consultant urological pathologists, with 12, 13 and 2 years’ experience post-FRCPath, were invited to assess the digital images for the selected slides on their usual DP workstations. All three had experience of DP of at least one year, and therefore did not require additional DP training prior to this study. Each pathologist was independently asked to make an assessment of the overall highest WHO/ISUP grade present in the selected sections from the cases, in accordance with the published guidance on assignment of WHO/ISUP grade, and this was documented in a spreadsheet. Initially the pathologists assessed the cases on a digital screen, and then subsequently repeated the assessment on the corresponding GS following a washout period of two weeks. A second assessment of the WHO/ISUP grade on the GS was carried out following a second washout period of at least two weeks. Two weeks was the selected time interval between reads (DP or GS) in accordance with the recommendation from the guideline for validating whole slide images for diagnostic purposes in pathology, issued by the College of American Pathology and Laboratory Quality Center [18]. The pathologists were blinded to the original histology report for the cases (which was not accessed at any point in the study except during the initial search for cases), and were blind to their prior assessments (DP and GS) and to those of the other two pathologists at the time of assessing. We did not attempt to revise the ISUP grade of the tumour in the original report on the basis of this study, and this would not have been appropriate given that only representative H&E slides had been assessed.

The WHO/ISUP grading was carried out in accordance with the published guidance [15], and this system has been routinely utilised for grading of CCRCC and PRCC by the urological pathologists in the department since 2013.

Statistical analysis on the data was carried out using a linear weighted Cohen’s kappa (Ƙ) coefficient for intraobserver comparisons (intra-rater comparisons) and Fleiss’ kappa (Ƙ) for interobserver comparisons (for all three pathologists). Calculations were made using standard formulae in Microsoft Excel.

## Results

The WHO/ISUP grade was independently determined by three consultant uropathologists on DP and on GS for a total of 60 cases of RCC (50 CCRCC and 10 PRCC). The assessment of WHO/ISUP grade on GS was performed on two separate occasions (termed read 1 and read 2) to provide an indication of intraobserver variability on GS assessment. The results are presented in Table 1.

**Table 1:** Summary of all WHO/ISUP grading for all three pathologists on DP and on GS read 1 and GS read 2. Cases 1–50 = clear cell renal cell carcinoma. Case 51–60 = papillary renal cell carcinoma

CASE	Nephrectomy (N) or partial nephrectomy (PN), or biopsy (B)	PATHOLOGIST A			PATHOLOGIST B			PATHOLOGIST C		
		**DP**	**GS1**	**GS2**	**DP**	**GS1**	**GS2**	**DP**	**GS1**	**GS2**
1	PN	3	3	3	2	2	3	2	3	3
2	N	3	3	3	3	3	3	3	3	3
3	PN	2	2	2	3	2	2	2	3	3
4	B	4	3	4	4	4	4	4	4	4
5	N	4	4	3	4	4	4	4	4	4
6	N	3	3	3	3	2	3	2	3	3
7	N	3	3	3	2	3	2	2	3	3
8	B	1	2	2	2	2	2	1	2	1
9	N	3	3	3	3	3	2	2	2	2
10	N	3	3	3	3	2	3	3	3	3
11	PN	3	3	3	3	3	2	3	3	3
12	N	4	4	4	4	4	4	4	4	4
13	PN	3	3	2	3	2	3	3	3	3
14	N	3	3	2	2	3	3	3	3	3
15	PN	2	3	2	2	2	2	2	3	3
16	N	4	4	3	3	4	4	3	3	3
17	N	2	3	3	2	2	2	2	2	3
18	PN	2	2	2	2	2	2	2	2	2
19	N	2	3	2	2	3	3	2	2	2
20	N	4	4	4	4	4	4	4	4	4
21	PN	2	2	2	2	2	2	2	2	2
22	PN	2	2	2	3	2	2	2	2	2
23	B	1	1	2	2	2	2	1	1	1
24	N	3	3	3	3	3	3	3	3	2
25	B	3	2	2	3	2	2	2	2	1
26	PN	3	3	3	4	4	4	3	3	3
27	PN	2	3	3	3	3	2	3	3	3
28	N	4	4	4	4	4	4	4	4	4
29	N	3	3	3	3	3	3	3	3	2
30	N	4	4	4	4	4	4	4	4	2
31	PN	3	3	3	3	3	3	3	3	3
32	PN	2	2	2	2	2	2	2	2	2
33	PN	2	2	2	3	2	2	1	2	2
34	N	3	3	3	3	2	3	3	3	3
35	PN	3	3	3	3	2	3	3	3	3
36	N	4	4	4	4	4	4	4	3	3
37	N	3	3	3	3	2	3	4	3	3
38	PN	2	3	2	2	2	2	2	2	2
39	N	2	2	2	2	2	2	2	2	2
40	PN	3	3	3	3	3	3	3	3	3
41	PN	3	3	3	2	2	2	2	3	3
42	N	3	3	3	3	2	2	3	3	3
43	PN	3	3	3	3	2	2	3	3	3
44	PN	3	3	3	3	3	3	3	3	3
45	PN	3	3	3	3	3	3	3	3	3
46	N	2	3	3	2	3	2	3	3	3
47	B	4	3	4	4	4	4	4	4	4
48	N	3	3	3	3	3	3	3	3	3
49	B	3	3	3	4	4	3	4	4	4
50	PN	3	3	3	3	3	3	3	3	3
51	B	2	2	2	2	2	2	1	2	1
52	PN	2	3	3	2	2	2	3	3	2
53	N	2	2	2	2	2	2	1	2	2
54	B	2	2	1	2	2	2	1	1	1
55	PN	3	3	3	3	2	2	3	3	3
56	N	3	2	2	2	2	2	1	3	2
57	N	2	2	2	1	2	2	2	2	2
58	PN	3	3	3	3	2	2	3	3	2
59	N	2	3	2	2	2	2	2	3	2
60	PN	3	3	3	3	2	2	3	3	3

*DP* digital pathology, *GS* glass slides

### Agreement of WHO/ISUP grading DP versus GS

**Fig. 1:**
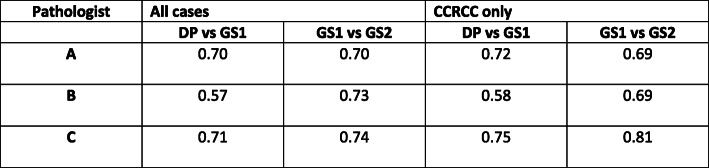
Cohen’s Kappa agreement for individual pathologists between DP and GS read 1 and for GS read 1 and GS read 2

Comparing WHO/ISUP grading on DP versus GS images (read 1) for all 60 cases, intraobserver agreement for the individual pathologists was 0.70, 0.57, 0.71 (Fig. 1). There was a marginal improvement in agreement for the grading of CCRCC only; 0.72, 0.58, 0.75.

The interobserver agreement on WHO/ISUP grade for all cases was 0.58 on digital, and 0.45 on glass (read 1), with slightly greater agreement for CCRCC alone (0.62 versus 0.50).

**Fig. 2:**
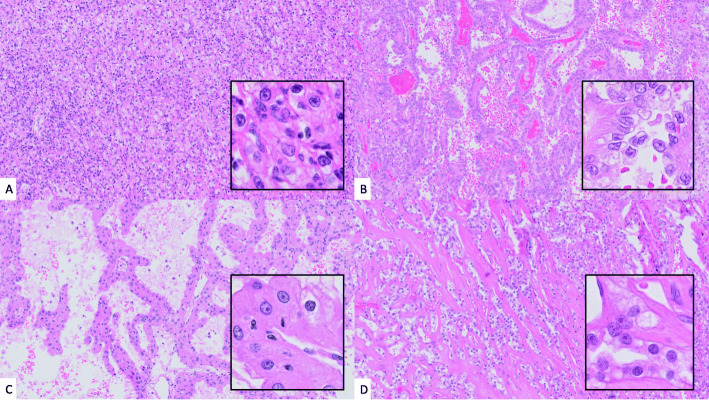
(**A**) CCRCC (H&E x10, inset H&E x40) case where all three pathologists agreed on ISUP grading (grade 3) on both DP and GS1 review. (**B**) PRCC (H&E x10, inset H&E x40) case which was upgraded by one pathologist by two grades (ISUP grade 1 on DP to 3 on GS1 review), and downgraded by one pathologist (ISUP grade 3 to 2). (**C**) CCRCC (H&E x10, inset H&E x40) case which was downgraded from ISUP grade 3 on DP to 2 on GS1 review by one pathologist, upgraded from ISUP 2 to 3 by one pathologist, and graded 3 by a third pathologist on both DP and GS1 review. (**D**) CCRCC (H&E x10, inset H&E x40) case which was upgraded from ISUP grade 2 on DP to 3 on GS1 review by two pathologists, and graded as ISUP 3 by a third pathologist on both DP and GS1 review. CCRCC = clear cell renal cell carcinoma, PRCC = papillary renal cell carcinoma, DP = digital pathology, GS = glass slide, ISUP = International Society of Urological Pathology.

We also looked at the grading agreement for each individual pathologist between DP and GS read 1 for each individual case, and overall this was 47/60 (78 %), 39/60 (65 %), and 46/60 (77 %) for pathologists A, B, and C respectively, and therefore 132/180 (73 %) overall intraobserver agreement in grade on DP vs. GS (read 1). There was very minimal improvement when limiting analysis to CCRCC alone; 40/50 (80 %), 33/50 (66 %), 40/50 (80 %). Of the 37 discrepancies in grade for CCRCC (DP grade versus GS grade for at least one pathologist), overall 17 were downgraded by one grade on GS review, and 20 were upgraded by one grade. None of the CCRCC cases were upgraded or downgraded by more than one grade. Of the 11 discrepancies in grade for PRCC (DP grade versus GS grade for at least one pathologist), overall 4 were downgraded by one grade on GS review, and 6 were upgraded by one grade. There was a single incidence of PRCC being upgraded by 2 grades on GS review (from grade 1 to 3, Fig. 2). In none of the cases (CCRCC or PRCC) did all three pathologists change the grade on GS review.

### Agreement of WHO/ISUP grading GS versus GS

Intraobserver variability on two separate GS reads was also examined to provide an indication of kappa agreement with which the DP versus GS kappa agreement could reasonably be compared (Fig. 1).

The kappa agreement for pathologists A, B and C between the two GS reads was 0.70, 0.73, 0.74 respectively, and for CCRCC alone it was 0.69, 0.69, 0.81 respectively.

For the individual pathologists, the comparative kappa scores for DP versus GS (read 1) and GS read 1 versus GS read 2 were 0.70 and 0.70 (pathologist A), 0.57 and 0.73 (pathologist B), and 0.71 and 0.74 (pathologist C).

The interobserver kappa score of agreement for GS read 1 was 0.45 versus 0.49 for GS read 2.

## Discussion

Multiple validation studies have demonstrated that DP is non-inferior to GS for histopathological diagnoses, however these studies have assessed overall diagnoses of cases rather than focussing on specific areas of discrepancy. Dysplasia has been identified within multiple of the studies as being an area of challenge for DP diagnosis, which is considered to be related to rendering of nuclear detail on DP, a factor that is confounded by scanning resolution (20x versus 40x) and/or compression artefacts. There is little within the published literature on validation of DP as to the precise cause of discrepancy between the assessment of nuclear detail on DP versus GS, beyond general statements that nuclear detail may be harder to appreciate on DP [2] and that the absence of facility to focus through the entire tissue section on DP (vs. GS) impacts the appreciation of nuclear chromatin [4]. A recent meta-analysis [19] noted that 57 % of clinically significant discordances between DP and light microscopy were of this nature. However, it is also recognised that assessment of dysplasia/grading is a source of discrepancy between pathologists on light microscopy, which may be a confounding factor. Furthermore, DP facilitates low power viewing of slides, and therefore there is also the potential for failure at low power to detect small areas of dysplasia (or higher grade areas) for further high power assessment [14].

Validation studies to date that include urological pathology specimens (summarised in Table 2), have consistently identified dysplasia related to urothelial specimens including grading of urothelial carcinoma as a recognised pitfall, but whilst grading of dysplasia in renal carcinoma is mentioned as a potential pitfall in one paper [14] this has not been specifically documented within the larger validation studies to date [2–13], nor within a small validation study devoted to urological specimens [20].

**Table 2:** Summary of major digital pathology validation studies which include urological specimens

AUTHOR	SCANNING SYSTEM EVALUATED	SCANNING MAGNIFICATION	STUDY METHODOLOGY	UROLOGICAL CASES INCLUDED IN STUDY	AREAS OF DISCORDANCE BETWEEN GLASS SLIDE (GS) READ AND DIGITAL PATHOLOGY (DP) READ FOR PURPOSE OF STUDY (EXCLUDING ORIGINAL DIAGNOSIS OR ADJUDICATED DIAGNOSIS WHERE GIVEN)
Campbell 2012 [5]	iScan®	x20	Single centre study 2 pathologists Digital diagnosis compared with original diagnoses (no washout period as the original glass slide diagnosis was the comparator), consensus diagnosis for discrepant cases	*N* = 6 Type not specified	Prostate biopsy (1 case) – benign (GS) vs. ASAP (DP) Bladder specimen (type not specified, 1 case) – suggestive of polypoid cystitis (GS) vs. PUNLMP (DP)
Bauer 2013 [3]	Aperio®ScanScope XT	x20	Multicentre study 2 pathologists Glass diagnosis followed by washout period of 1 year, then either digital or GS review, consensus diagnosis for discrepant cases	Type not specified	Prostate specimens (type not specified, 6 cases) – Gleason grading (x3 cases), benign (GS) vs. HG PIN (DP), PIN (GS) vs. benign (DP)
Al-Janabi 2014 [19]	Not specified	x20	Single centre study 2 pathologists Digital diagnosis compared with original diagnoses (each pathologist reviewed only the GS cases they originally diagnosed), washout period minimum 6 months. The original glass slide diagnosis was the comparator, consensus diagnosis for discrepant cases	*N* = 100 Urinary system only Kidney = 50 (48 cases were medical renal disease diagnoses, 2 surgical diagnoses) Bladder = 43 Ureter = 1 Urethra = 6	Excluding the medical renal cases Bladder specimens (5 cases) – Grade 3 PUC, non-invasive (GS) vs. grade 3 PUC with lamina propria invasion (DP), benign no abnormality (GS) vs. chronic inflammation (DP), grade 3 PUC, suspicious for invasion (GS) vs. grade 3 PUC with lamina propria invasion and CIS (DP), & 2 cases with minor descriptive discrepancy only
Snead 2016 [8]	Omnyx® VL4	x40	Single centre study 17 pathologists Glass slide diagnosis, followed by washout period of 21 days, before digital read (33 % cases reported on GS and digital by the same pathologist, and 66 % by two different pathologists), consensus diagnosis for discrepant cases	*N* = 242 Type not specified	Penile biopsy with HPV changes and atypia (GS) vs. PeIN (DP) Prostate biopsies (2 cases) – Gleason grading (pattern 4 vs. 3), suspicious for malignancy (GS) vs. benign (DP) Urothelial biopsies (3 cases) – Urothelial carcinoma grade 1 LG (GS) vs. grade 2 HG (DP), urothelial carcinoma with no CIS (GS) vs. urothelial carcinoma with CIS (DP), non-invasive urothelial carcinoma (GS) vs. urothelial carcinoma with early invasion (DP)
Tabata 2017 [9]	PhilipsIntelliSite® Ultrafast scanner Leica Biosystems® Aperio ®AT2 scanner Hamamatsu® Nanozoomer® 2.0-HT C9600-13 Hamamatsu® NanoZoomer® 2.0-RS C10730-13 CLARO FINO	Variable, x20 and x40	Multicentre study 9 pathologists Each pathologist carried out digital and glass slide reads on each case, washout period minimum of 14 days, consensus diagnosis for discrepant cases	*N* = 66	Prostate specimen (type not specified, 1 case) – benign (GS) vs. atypical glands (DP) Genitourinary organ (not specified, 1 case) – erosive mucosa without malignancy (GS) vs. erosive mucosa possible for malignancy (DP)
Mukhopadhyay 2018 [7]	Philips IntelliSite® Pathology Solution	Not specified	Multicentre study 16 pathologists Each pathologist carried out digital and glass slide reads on each case, washout period minimum of 16 days, reference standard = original GS diagnosis, with adjudication for discrepant cases	*N* = 448 Urinary bladder = 99 Prostate = 299 Kidney, neoplastic = 50	Kidney, neoplastic (2 cases) – papillary RCC (GS) vs. metanephric adenoma (DP), HG papillary urothelial carcinoma (GS) vs. LG urothelial carcinoma (DP) Urinary bladder (not otherwise specified) (18 cases) – cystitis with reactive atypia (GS) vs. HG CIS (DP), HG non-invasive PUC (GS) vs. HG PUC with lamina propria invasion (DP), HG PUC without invasion (GS) vs. LG PUC non-invasive (DP), LG PUC non-invasive (GS) vs. LG PUC with invasion (DP), cystitis with hyperplasia (GS) vs. flat HG dysplasia (DP), HG urothelial carcinoma (GS) vs. fibrosis, benign (DP), tissue highly suspicious for invasive SCC (GS) vs. mildly atypical squamous epithelium, favour squamous metaplasia (DP), HG urothelial carcinoma invading through bladder wall into perivesical soft tissue (GS) vs. benign (DP), cystitis with mucosal ulceration with reactive atypia (GS) vs. myoinvasive HG urothelial carcinoma (DP), cystitis with granulomatous features and reactive atypia (GS) vs. myoinvasive HG urothelial carcinoma (DP), CIS (GS) vs. cystitis (DP), HG PUC non-invasive (GS) vs. HG PUC with lamina propria invasion (DP), cystitis with reactive atypia (GS) vs. CIS (DP), CIS (GS) vs. HG urothelial carcinoma with lamina propria invasion (DP), atypical urothelium (GS) vs. benign (DP), CIS (GS) vs. inflammation (DP), CIS (GS) vs. inflammation (DP), CIS (GS) vs. inflammation (DP)
Vodovnik 2018 [13]	Aperio® ScanScope® AT Turbo	x20	Single centre study 1 pathologist Digital diagnosis compared with original diagnosis (the pathologist had reported the cases on both GS and DP), washout period 6 months.	*N* = 75	Prostatic adenocarcinoma grading Gleason 9 (5 + 4) (GS) vs. Gleason 9 (4 + 5) (DP) UC HG grade 2–3 (GS) vs. UC HG grade 2 (DP)
Borowsky 2020 [4]	Leica Biosystems® Aperio® AT2 DX system	x20	Multicentre study 19 pathologists Each pathologist carried out digital and glass slide reads on each case, washout period minimum of 31 days, reference standard = original GS diagnosis, with adjudication for discrepant cases	*N* = 447 Urinary bladder = 100 Prostate = 300 Kidney, neoplastic = 47	Not specified, although comment that urinary bladder biopsies showed the highest major discrepancy rate
Hanna 2020 [11]	Leica Biosystems® Aperio® GT450	x40	Single centre study 12 pathologists (2 reporting GU cases) Each pathologist carried out digital and glass slide reads on each case, the digital read was done remotely via a virtual private network (VPN), and the GS read was done on site in the hospital department with a mean interval of 2 days. Reference standard = GS diagnosis with adjudication for discordant cases.	718 slides = 108 cases in total across specialties, and for GU the following specimens; Prostate = 151 Bladder = 28 Lymph nodes = 10 Kidney = 9 Urethra = 6 Testis = 3 Ureter = 1 Adrenal = 1 Other = 10	No major or minor discordances
Rao 2021 [12]	Ventana® DP200	x20 (x40 scanning available on request)	Single centre study 18 pathologists Study looked at concordance between digital sign out of cases remotely (from home) with blinded re-review of cases after a minimum 2 week interval. Concordance adjudicated by a referee pathologist not participating in the sign out study. Blind consensus diagnosis established for discordant diagnoses.	*N* = 25 (1 of which was deferred to glass) 47 parts, 74 slides; Urinary bladder, ureteric orifice = 24 Kidney = 5 Penis = 2 Prostate = 12 Iliac fossa = 1 Lung = 1 Endometrium = 1 Rectum = 1	Urinary bladder (1 case, TURBT) HG PUC T1 (GS) vs. HG PUC Ta (DP)

*GS* glass slides, *DP* digital pathology, *ASAP* atypical small acinar proliferation, *PUNLMP* papillary urothelial neoplasm of low malignant potential, *PIN* prostatic intraepithelial neoplasia, *PUC* papillary urothelial carcinoma, *CIS* carcinoma in situ, *HPV* human papilloma virus, *PeIN* penile intraepithelial neoplasia, *LG* WHO 2004 low grade, *HG* WHO 2004 high grade, *RCC* renal cell carcinoma, *SCC* squamous cell carcinoma, *GU* genitourinary, *TURBT* transurethral resection of bladder tumour

Given this, and the clinical importance of WHO/ISUP grading in CCRCC and PRCC, we sought specifically to assess the concordance of WHO/ISUP grading on DP and GS. In so doing, we also determined the intraobserver and interobserver agreement for assignment of WHO/ISUP grade.

Our results show that overall the assessment of WHO/ISUP grade on DP is non-inferior to that on GS. The concordance of WHO/ISUP grading on DP and GS was 65 to 78 % across the three pathologists, though it is noted that the individual kappa scores could be regarded only as ‘moderate to substantial‘. The concordance was slightly improved if the analysis was limited to CCRCC cases. Importantly the individual kappa agreement between DP and GS (read 1) and the two GS reads was almost identical for two of the pathologists (Ƙ = 0.70 and 0.70, Ƙ= 0.71 and 0.74 respectively), and marginally different for the third pathologist (Ƙ = 0.57 vs 0.73), which also suggests the non-inferiority of DP for WHO/ISUP grading.

Importantly, our results have demonstrated that there does not appear to be a tendency either way to over or undergrade either CCRCC or PRCC on DP compared with GS; 17 CCRCC grades (across all three pathologists, i.e. out of 180 grading events) being downgraded by one grade on glass review, and 20 upgraded by one grade, and 4 PRCC grades being downgraded by one grade on glass review, and 6 upgraded by one grade. A single PRCC grade was upgraded from 1 to 3 on GS review (one pathologist). This is reassuring given the significance of the tumour grade for prognostication and management planning, including for example, within the Leibovich scoring system [17]. However, we would advocate that in spite of the reassurance proffered by these results, that pathologists retain a low threshold for seeking further opinion when they are uncertain about assignment of grade as, particularly for CCRCC, this can make a considerable difference to the risk group a patient falls into [17], and their subsequent management.

Whilst the intraobserver agreement between DP and GS is considered to be the most appropriate method to evidence whether DP performance is as reliable as conventional microscopy [2, 18], we sought also to determine the overall interobserver agreement on WHO/ISUP grade. Across all cases the interobserver kappa scores of agreement on grade were 0.58 on DP, 0.45 on GS read 1, and 0.49 for GS read 2. When the analysis was restricted to CCRCC, the interobserver kappa agreement was minimally different; 0.62 on DP, 0.50 on GS read 1, 0.48 on GS read 2. These figures suggest marginal greater agreement of interobserver kappa score for DP over GS, although this is a small study.

Given that WHO/ISUP grading conveys important prognostic information as to the likely behaviour of CCRCC or PRCC, influencing the patient follow-up protocol and potentially clinical trial entry, it is noteworthy that there is a lack of published literature on the reproducibility of the WHO/ISUP grade, although it is commented upon by some authors that there is recognised interobserver variability [21]. To the best of the authors’ knowledge, this is the first study formally to report on reproducibility of WHO/ISUP grade, either between pathologists or on separate sittings for the same pathologist.

Prior to the ISUP grading system the issues of reproducibility of the Fuhrman grading system were recognised; for example, one study reported only a low to moderate level of interobserver agreement on Fuhrman grading of RCC (mean Ƙ value 0.22) [22]. These difficulties in reproducibility were felt in part to be due to the multifactorial nature of this grading system, which included nuclear diameter, nuclear shape, and nucleolar prominence [23]. This led in part to the proposal of a new system based upon nucleolar grading [23], and this subsequent ‘ISUP grading system’ [16] was then designated with minor modifications as the WHO/ISUP grading system, [15]. However, the reproducibility of this new grading system is not specifically mentioned or indeed assessed in papers validating it [24–27].

We have shown in this study that the intraobserver reproducibility of WHO/ISUP grading of CCRCC and PRCC by three specialist urological pathologists can be considered substantial, and consistently so when comparing assessment on glass slides. Interobserver reproducibility is however only moderate, although it is greater than the reported reproducibility of Fuhrman grade [22]. There does appear to be slightly greater interobserver reproducibility of grade when assessed on DP in comparison to GS, something that has in fact been previously postulated [21], however this is a small study. This observation warrants further study and potentially lends itself to automated assessment using artificial intelligence (AI), which may further improve consistency of grading in future. Indeed, a recently published study reported the development of a deep learning model to determine the grade of RCC, and suggested that the categorical accuracy for predicting tumour grade (Fuhrman) using this model was 98.4 % [28]. Such tools may be of value in improving grading accuracy in future.

## Conclusions

We have demonstrated in this validation study that DP is non-inferior to GS in terms of assessment of WHO/ISUP grading in CCRCC or PRCC. However assessment of nuclear detail is a recognised area of potential challenge in assessment in DP and, given that WHO/ISUP grading relies upon nucleolar features pathologists should remain aware that this is a potential pitfall.

The reproducibility of WHO/ISUP grade is of direct clinical relevance, and whilst we have demonstrated that this appears to be moderate to substantial at both intraobserver and interobserver level, it would seem that DP may potentially facilitate greater consistency in grading. AI tools to automate grading of RCC may offer a further means to improve reproducibility.

## Data Availability

All data generated or analysed during this study are included in this published article.

## Abbreviations

WHO: World Health Organisation
ISUP: International Society of Urological Pathology
RCC: Renal cell carcinoma
CCRCC: Clear cell renal cell carcinoma
PRCC: Papillary renal cell carcinoma
DP: Digital pathology
GS: Glass slide
FRCPath: Fellowship of the Royal College of Pathologists
AI: Artificial intelligence
